# Have you looked for “stranger things” in your automated PET dose dispensing system? A process and operators qualification scheme

**DOI:** 10.1186/s41181-019-0061-8

**Published:** 2019-06-13

**Authors:** Tristan Martin, Anaïs Moyon, Cyril Fersing, Evan Terrier, Aude Gouillet, Fabienne Giraud, Benjamin Guillet, Philippe Garrigue

**Affiliations:** 1 Radiopharmacie Hôpital Nord AP-HM, Marseille, France; 2 Radiopharmacie Hôpital Timone AP-HM, Marseille, France; 3 CERIMED, CNRS 2012, Aix-Marseille Université, Marseille, France; 4 C2VN, INRA 1260, INSERM 1263, Aix-Marseille Université, Marseille, France

**Keywords:** PET, Radiopharmaceuticals, Qualification, Dispensing system

## Abstract

**Background:**

Many nuclear medicine departments have equipped themselves with automated dispensing systems (ADS) for PET radiopharmaceuticals, in both the operators’ and the patients’ interests. Whether partially or fully automated, to date there is no marketed ADS representing a true “closed-system”. Despite the sterile, injectable nature of ready-to-use radiopharmaceutical drug solutions manipulated by these systems, neither manufacturer’s recommendation nor literature report was found about specific qualification of the process’ sterility, or about operators’ qualification on these dispensing systems. We set up a master plan validation in a radiopharmacy equipped with Trasis Unidose®, including: 1) monthly process-simulating media-fill tests and microbiological contamination assessments of the ADS surfaces; 2) initial and periodic qualification of the operators. The microbiological qualification consisted in surface biocontamination assessment on critical zones with Tryptic-Soy agar. The operator qualification consisted in the evaluation of the operators’ knowledge and skills for using the ADS.

**Results:**

This study highlighted a minor, handborne microbiological contamination on our first assessment, that corrective actions solved. We therefore decided to brief our operators once a month on microbiological control results, hygiene and good practices, with the support of the present case illustrating the biodecontamination efficiency.

**Conclusions:**

As the automation of PET monodose conditioning process still implies human intervention for material preparation and manual biodecontamination, this study illustrates that initial and periodic qualification of the environment and the conditioning process of ADS, including microbiological qualification and operators’ qualification, are needed to meet specifications.

## Background

The expanded use of positron-emission tomography (PET) and the intercurrent market authorizations of new PET radiopharmaceuticals implied optimization of their dispensing and administration, in both the operators’ and the patients’ interests. Since many evidences showed substantial benefits in radioprotection for the operators in terms of dosimetry, many nuclear medicine departments have equipped themselves with automated dispensing systems (ADS) (Covens et al. 2010). Most of the validations found in the literature consist in monitoring physico-medical parameters such as operator dosimetry and accuracy and/or precision of dose activities measurements through dose calibrators (Lecchi et al. 2012; O’Doherty et al. 2014). Whether partially or fully automated, to date there is no marketed ADS representing a true “closed-system”. For instance, the Trasis Unidose® features a cartridge-filling step which is automatically operated under an open, virtual ISO4.8 system, whose performance is obviously critical for maintaining the microbiological quality of the drug solution. Furthermore, in many radiopharmacies and nuclear medicine departments, the ADS is installed in a non-classified room. Still, despite the sterile, injectable nature of ready-to-use radiopharmaceutical drug solutions manipulated by these systems, neither manufacturer’s recommendation nor literature report was found about specific qualification of the process’ sterility, or about operators’ qualification on these dispensing systems. The basic *International Standard Organization* (ISO) standards for controlled environment, and the *Good Manufacturing Practices* (GMP) for production of injectable solutions, still apply to these ADS (ISO n.d.; EudraLex n.d.). A preliminary risk analysis was therefore led in our department prior to the ADS setup and highlighted the microbiological contamination risk could be significant.

The aim of this work was to establish a practical, methodological guideline to help the dozens of Nuclear Medicine centers already equipped with such an ADS (and those intending to) for their routine microbiological qualification and operators’ qualification, based on our practical experience.

Inspired by guidance for qualification of small-scale PET radiopharmaceutical synthesis modules, and in respect with the Pharmaceutical Inspection Co-operation Scheme (PIC/S), the *European Pharmacopeia* and the Food and Drug Administration (FDA) guidance, we set up a master plan validation in a radiopharmacy equipped with Trasis Unidose®, including: 1) monthly specific process-simulating media-fill tests (MFT) and microbiological contamination assessments of our ADS surfaces; 2) initial qualification and yearly requalification of the ADS operators (Aerts et al. 2014; PIC/S n.d.; Food and Drug Administration CDER CGMP 2012). The first six-month follow-up period already showed the importance of implementing and long-term pursuing such a process to ensure the continuous performances of the ADS and good practices application.

## Methods

### Description of equipment

Qualification was performed on a Unidose® dispenser (Trasis) placed in an adapted shielded cabinet equipped with gloves and a shielded glass oculus (Trasis). The cabinet features a main ISO7 compartment hosting the ADS. The multidose PET radiopharmaceutical drug is contained in a closed reservoir, part of the ADS. The individual dose is obtained by cartridge filling, that is weight-controlled operated under an open, local ISO4.8 zone ensured by a High Efficiency Particulate Air (HEPA) filter, inside the ISO7 compartment. During this critical step, the radiopharmaceutical drug solution leaks from a 0.22 μm sterile filter and fills the open cartridge, which is hereafter capped in the same ISO4.8 zone, then measured in a dose calibrator and finally dispensed.

### Microbiological validation - surface biocontamination assessment

**Fig. 1:**
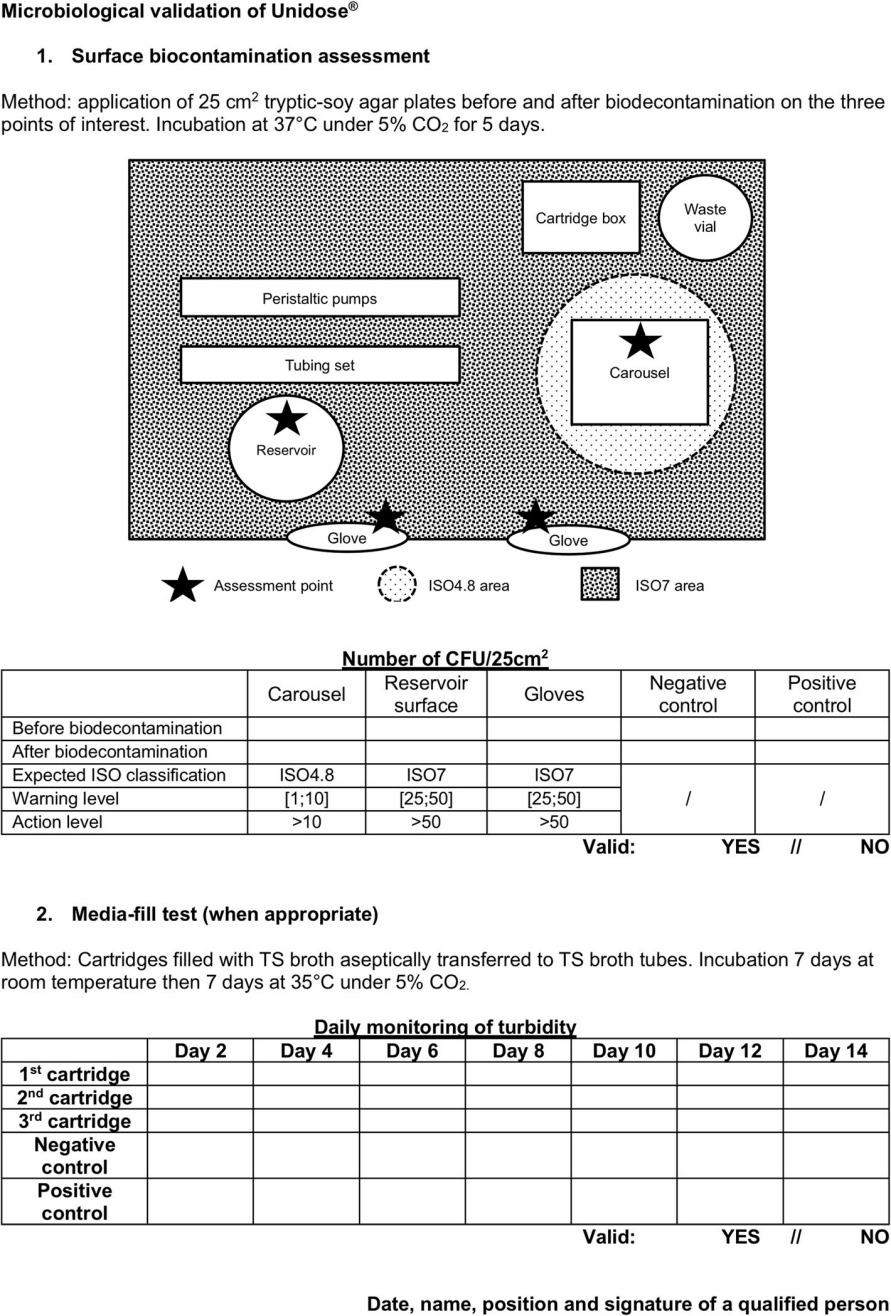
Datasheet for microbiological qualification of Unidose®

Microbiological contamination of surfaces was assessed at the end of a routine PET session on different points (Fig. 1): the carousel in the filling zone (ISO4.8), the gloves (ISO7) and the reservoir surface (ISO7). Through the application of 25cm^2^ tryptic-soy (TS) agar (Becton-Dickinson) before and after biodecontamination following internal procedures (intensive and precautious cleaning with a disinfectant detergent solution based on a N-(3-aminopropyl)-N-dodecylpropane-1, 3-diamine, didecyldimethylammonium chloride and propan-2-ol mixture), we characterized the surface biocontamination. Agars were incubated at 37 °C under 5% CO_2_ for 5 days and colonies were then counted (CFU/25cm^2^). Further identification was realized by our Microbiology Department. Positive and negative control agars were prepared and incubated simultaneously. Surface biocontamination assessment was operated by a qualified radiopharmacist, repeated three times each session and followed-up monthly for 6 months. Results were expressed as mean ± s_d_ CFU/25 cm^2^.

### Microbiological validation - media fill tests

MFTs were carried out to evaluate the whole filling process sterility at the end of a routine PET session. To achieve those adapted MFT, a sealed, sterile TS broth vial (Becton-Dickinson) was put in place of the leaded multidose radiopharmaceutical vial, then the dispensing process was initiated. The resulting cartridges were aseptically transferred to TS broth tubes (Becton-Dickinson) and according to the *European Pharmacopoeia* specifications, placed for incubation 7 days at room temperature and then 7 days at 37 °C under 5% CO_2_, with daily monitoring of turbidity. Positive and negative control solutions were prepared and incubated simultaneously. MFT was operated by a qualified radiopharmacist, repeated three times each session and followed-up monthly for 6 months. Results were expressed as mean ± s_d_ number of contaminated tubes.

### Operator qualification (OpQ)

**Fig. 2:**
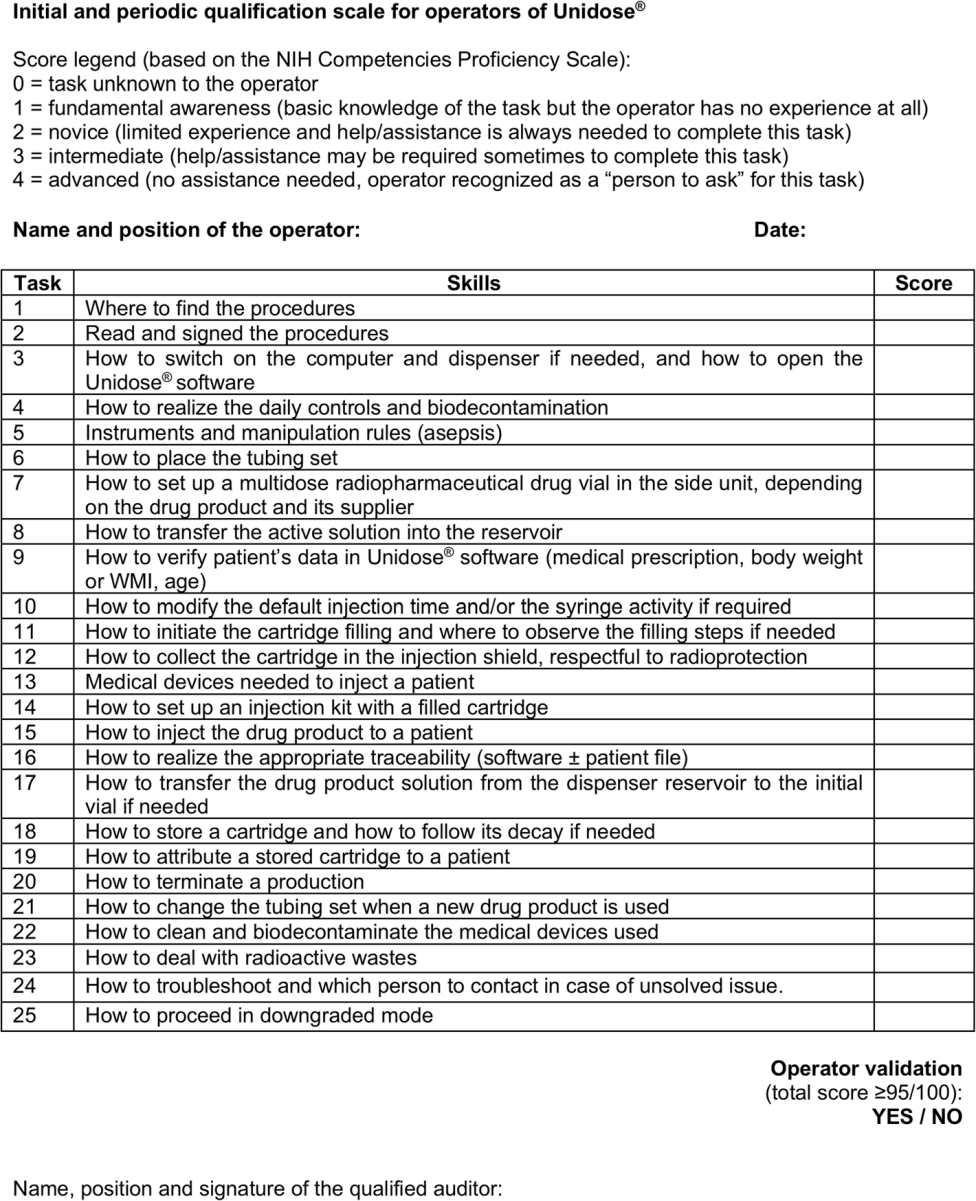
Initial and periodic qualification scale for operators of Unidose®

Operators were individually trained and evaluated by one and only qualified radiopharmacist. A qualification scale inspired from the NIH Competencies Proficiency Scale (Fig. 2) was established and focused on evaluating the operators’ knowledge of the equipment, materials, software and accessories, their knowledge of our internal procedures regarding the ADS cleaning, setup, single dose dispensing process, backup mode, radioprotection, and their ability to apply these procedures during a PET session. The operators were considered as qualified if the score was equal to or superior than 95/100. They were briefed on our procedures and general good practices when appropriate.

### Statistics

Surface microbiological contamination data were submitted to a two-tailed Wilcoxon matched-pairs signed rank test. *P* ≤ 0.05 indicated significance.

## Results

### Microbiological validation - surface qualification

**Fig. 3:**
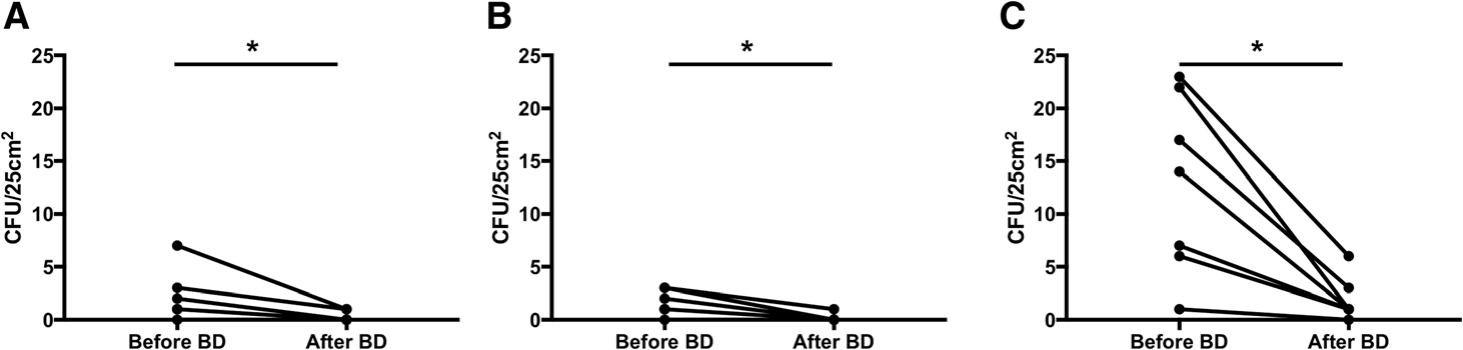
The importance of an adequate biodecontamination on microbiological qualification. Microbiological qualification results on 3 areas of interest: the carousel with ISO4.8 specifications (**a**), the reservoir surface (**b**) and the gloves (**c**) both with ISO7 specifications, before and after biodecontamination (BD). *P < 0.05, *n* = 7; two-tailed Wilcoxon matched-pairs signed rank test.

A minor microbiological contamination was found at the first assessment on TS agars sampled from the carousel (7.1 ± 1.5 CFU/25cm^2^) and was identified as classical hand-borne microflora (*Micrococcus luteus* and *Staphylococcus sp.*). Investigating for the biocontamination origin, the cap used for daily sterile filter sealing tests was found contaminated by the same microflora (13.2 ± 3.8 CFU/25cm^2^). We consequently adapted our daily biodecontamination procedure to include this instrument and renewed advice to the operators on this procedure knowledge and general hygiene procedures. Since then, all the agars realized on 7 independent sessions have stayed in line with ISO classification recommendations on the three areas of interest. The modified biodecontamination procedure significantly reduced microbiological contamination on the carousel (2.14 ± 2.34 CFU/25cm^2^ before, 0.286 ± 0.49 CFU/25cm^2^ after biodecontamination, *P* = 0.0312, *n* = 7), on the reservoir surface (1.57 ± 1.13 CFU/25cm^2^ before, 0.14 ± 0.39 CFU/25cm^2^ after biodecontamination, *P* = 0.0312, *n* = 7) and on the gloves (12.9 ± 8.43 CFU/25cm^2^ before, 1.86 ± 2.03 CFU/25cm^2^ after biodecontamination, *P* = 0.0156, *n* = 7), as shown in Fig. 3.

### Microbiological validation – media-fill tests

No microbiological contamination was detected in any TS tube. The media fill test resulted in clear, sterile cartridges (*n* = 7), validating the process sterility.

### Operator qualification

Two radiopharmacists, 3 pharmacy residents, 1 pharmacy technician and 13 technologists underwent OpQ by a qualified, experienced radiopharmacist. At the end of OpQ, the staff was specifically made aware of daily biodecontamination importance and general hygiene procedures. The staff successfully qualified with a mean score of 97 ± 2/100, reflecting its adequate knowledge and understanding of procedures and its ability to apply these with only a few or no assistance needed at all following trainings and briefings. As OpQ will be repeated on a yearly basis, personal results are archived to enable a follow-up of the competencies from a year to another.

## Discussion

In most European countries, it is the radiopharmacist’s responsibility to ensure aseptic conditions for preparing or manufacturing injectable drug products and for their dispensation, including radiopharmaceutical drugs whether the manufacturing/preparation process is automated or not (Decristoforo and Patt 2016). Regarding PET ADS, a complete initial qualification is classically carried out by the manufacturer or the distributor once the equipment is installed (Table 1): in our department, the initial performance qualification was negative for microbiological contamination of air (< 1 UFC/m^3^) and surfaces (<1UFC/25cm^2^). Still, the annual maintenance includes particular qualification, but excludes microbiological qualification with no specific recommendation from the manufacturer.

**Table 1:** Initial Trasis Unidose® IQ/OQ/PQ

IQ	OQ	PQ
• Setup check-list • Network configuration • Trainings	• Labels data consistency • Air bubble size • Cartridge closure	• Requested activity vs filled activity precision • Internal dose calibrators • precision vs external calibrated dose calibrator • Particular qualification • Microbiological qualification (externalized) • External dose rate • PC/Unidose bridge

Legend: This table provides summarized data about installation qualification (IQ), operational qualification (OQ) and performance qualification (PQ) initially performed by the supplier.

After setting up our internal qualification plan, we highlighted a minor microbiological contamination on our first assessment, that corrective actions solved and made finally the results conform to GMP. We therefore decided to brief our operators once a month on microbiological control results, hygiene and good practices, with the support of the present case illustrating the biodecontamination efficiency.

According to GMP, active air monitoring should be performed additionally to surface monitoring to complete the microbiological qualification. Yet, active air monitoring in Trasis Unidose® is very limited as the space for positioning an air impactor to sample the classified areas, is very limited itself. The manufacturer comes with its own specifically-designed air impactor system handler for the planned yearly maintenance, but such a device is not sold with the ADS so self-qualification seems hazardous when positioning classical air impactor systems. That’s precisely the reason why we decided not to recommend such a control, but rather to introduce an adapted MFT for the ADS: although not designed to assess any ISO classification area, this MFT not only validates the open-system step for cartridge filling, but also validates the whole-process of cartridge-manufacturing sterility, complementary to surface monitoring.

More generally for any ADS, a monthly microbiological control of surfaces and instruments should be recommended, as well as a media-fill test that should be repeated after each maintenance and/or modification of the dispensing process, in line with the recent EANM guidelines on validation and qualification of processes and operators (Todde et al. 2017).

An initial and yearly operator’s qualification should be established including the whole ADS applications. Regular training and information should be dispensed to the operators, regarding the importance of properly carrying out the biodecontamination of surfaces and instruments.

## Conclusions

The automation of PET monodose conditioning process still implies human intervention for material preparation and manual biodecontamination. This study illustrates that initial and periodic qualification of the environment and the conditioning process of ADS, including regular microbiological qualification and operators’ qualification, are needed to meet specifications and answer quality standards, in the interest of the patient’s safety.

## Data Availability

All data generated or analyzed during this study are included in this published article.

## Abbreviations

ADS: Automated dispensing system
CFU: Colony-forming unit
EANM: European Association of Nuclear Medicine
FDA: Food and Drug Administration
GMP: Good Manufacturing Practices
HEPA: High Efficiency Particulate Air
IQ: Installation qualification
ISO: International Organization for Standardization
MFT: Media-fill test
NIH: National Institutes of Health
OpQ: Operator qualification
OQ: Operational qualification
PET: Positron-emitting tomography
PIC/S: Pharmaceutical Inspection Co-operation Scheme
PQ: Performance qualification
TS: Tryptic-soy
